# Diagnostic utility of a one-item question to screen for depressive disorders: results from the KORA F3 study

**DOI:** 10.1186/1471-2296-14-198

**Published:** 2013-12-23

**Authors:** Eva Blozik, Martin Scherer, Maria E Lacruz, Karl-Heinz Ladwig

**Affiliations:** 1 Department of Primary Medical Care, University Medical Center Hamburg-Eppendorf, Hamburg, Germany; 2 Institute of Social Medicine, University of Lübeck, Lübeck, Germany; 3 Helmholtz Zentrum München, German Research Center for Environmental Health, Institute of Epidemiology-II, Neuherberg, Germany; 4 Department of Psychosomatic Medicine and Psychotherapy, Klinikum rechts der Isar, Technische Universität München, Munich, Germany

## Abstract

**Background:**

Screening for depressive disorders in the general adult population is recommended, however, it is unclear which instruments combine user friendliness and diagnostic utility. We evaluated the test performance of a yes/no single item screener for depressive disorders (“Have you felt depressed or sad much of the time in the past year?”) in comparison to the depressive disorder module of the Patient Health Questionnaire (PHQ-9).

**Methods:**

Data from 3184 participants of the population-based KORA F3 survey in Augsburg/ Germany were used to analyse sensitivity, specificity, ROC area, positive likelihood ratio (LR+), negative likelihood ratio (LR-), positive predictive value (PPV), and negative predictive value (NPV) of the single item screener in comparison with “depressive mood” and “major depressive disorder” defined according to PHQ-9 (both interviewer-administered versions).

**Results:**

In comparison to PHQ-9 “depressive mood”, sensitivity was low (46%) with an excellent specificity (94%), (PPV 76%; NPV 82%; LR + 8.04; LR- .572, ROC area .702). When using the more conservative definition for “major depressive disorder”, sensitivity increased to 83% with a specificity of 88%. The PPV under the conservative definition was low (32%), but NPV was 99% (LR + 6.65; LR- .196; ROC area .852). Results varied across age groups and between males and females.

**Conclusions:**

The single item screener is able to moderately decrease post-test probability of major depressive disorders and to identify populations that should undergo additional, more detailed evaluation for depression. It may have limited utility in combination with additional screening tests or for selection of at-risk populations, but cannot be recommended for routine use as a screening tool in clinical practice.

## Background

Depressive disorders are a major burden for the healthcare systems worldwide leading to loss of productivity, functional decline, and increased mortality [1-6]. The daily functioning and overall health of patients with depression can be improved when patients receive appropriate therapies [7,8]. Screening alone does not improve the health of patients with undiagnosed depressive disorders [9-12] but screening combined with patient-support programs, such as regular nurse follow-ups and close monitoring of adherence to therapy, seems to be useful [13]. Therefore, the U.S. Preventive Services Task Force recommends screening for depressive disorders in the general adult population when there are staff-assisted depression care supports in place to assure accurate diagnosis, effective treatment, and follow-up [14]. Additionally, screening for depressive disorders is recommended in populations at risk such as those with a family or personal history of depressive disorders, multiple medical problems, unexplained physical symptoms, chronic pain, or use of medical services that is more frequent than expected even if no depression care supports are available [15].

For screening purposes, different instruments exist [16]. Administering and evaluating comparatively long screening instruments can be time-consuming and it may thus be difficult to implement them in busy clinical settings [17]. Simple tests focusing explicitly on depressive disorders and without the need for additional computation on the clinician’s side seem to have the highest probability that this information is integrated into the clinical decision-making process [18]. In the context of comprehensive research evaluations long instruments may increase respondent burden [19]. This is why research teams searching for the shortest possible measure proposed and evaluated screeners consisting of one or two items [20]. Williams et al. presented a simple and easy-to-administer single item question (“Have you felt depressed or sad much of the time in the past year?”) and reported good sensitivity and less specificity as compared to the Center for Epidemiologic Studies Depression Screen (CES-D) [21] using a diagnostic SCID interview (Structured Clinical Interview for Diagnostic and Statistical Manual of Mental Disorders) as the criterion (85% vs 88% and 66% vs 75%, respectively) [11]. In contrast, Corson et al. found this single item to be specific (88%) but less sensitive (78%) when using the 9-item Patient Health Questionnaire (PHQ-9) [22] algorithm for major depression as the reference standard [20].

Given these discrepancies and given the fact that the previous studies were conducted in very specific study populations (predominantly female Hispanics or veterans in the USA), this study evaluates measures of test performance of the Williams et al. single-item screener in comparison to PHQ-9 in a population-based sample of adults from Germany. The aim of this study is to conclude on the utility of this single item screener to screen for depression in the general population.

## Methods

### Study design and subjects

The data stem from the city of Augsburg (Bavaria, Germany) and from surrounding districts covering about 600,000 inhabitants drawn from mixed urban and rural areas whose demographic and socioeconomic characteristics roughly reflect those of the average middle European population in general. The present analysis investigates data from the population-based KORA F3 survey conducted in 2004/05 within the framework of the ongoing KORA project (Cooperative Health Research in the Augsburg Region, Germany), a research platform for population-based health research [23]. The KORA F3 survey is a follow-up survey to the MONICA S3 survey conducted in 1994/95—at that time one cooperative centre within the worldwide WHO MONICA (Monitoring Trends and Determinants on Cardiovascular Diseases) project investigating the general and cardiovascular health of diverse populations. For the MONICA S3 survey, a stratified random representative sample of 6481 eligible subjects was drawn in 1994/95 from the population, of whom a total of 4856 subjects (response rate: 74.9%) participated in the S3 baseline survey. By the F3 follow-up study one decade later (2004/05), a total of 405 (8%) subjects had died. Furthermore, subjects were considered ineligible for inclusion in the F3 follow-up survey if they lived too far outside the study region or were completely lost to follow-up (n = 222, 5%), or had demanded deletion of their address data (n = 270, 6%). Of the remaining 3959 eligible subjects, 161 could not be contacted, 295 were unable to come because they were too ill, and 497 were not willing to participate, resulting in an interim total of 3006 participants in the F3 follow-up survey (response rate: 76% of S3 participants). Furthermore, additional efforts were made to reach those 1300 eligible subjects from the original S3 sampling frame who had not participated in the S3 baseline survey. Thus, another 178 (14%) participated in the present KORA F3 study, for a total sample size of 3184 (overall response rate: 49.12%). Written informed consent was obtained from each study participant and the study was approved by the ethics committee of the Bavarian Medical Association.

### Instruments

All participants underwent a standardized face-to face interview including the Patient Health Questionnaire and the single item screener and an extensive medical examination. The interviews were performed by experienced study nurses at the KORA Study Centre, Augsburg. Before start of the study, they received an extended training program and were certified thereafter. All interviews were taped and subjected to a routine quality assessment in the KORA data centre to avoid bias. At study halftime, all interviewers were recertified. Depression was assessed in an interview version of the 9 item depression module of the Patient Health Questionnaire (PHQ-9) [24]. Patients rate the frequency of symptoms of depression over the past 2 weeks on an ordinal scale (0 = not at all, 1 = several days, 2 = more than half the days, 3 = nearly every day). The 9 items are based on the 9 DSM-IV criteria for the diagnosis of depression. The total score ranges from 0 to 27. In order to be congruent with the DSM-IV criteria, the algorithm developed and validated by Spitzer et al. was used for classification: “Major depressive disorder” was defined as having at least five questions answered with “more than half the time in the past two weeks”, of which at least one of the first two questions (little interest in doing things, feeling depressed) had to be included. Participants were labelled to have “depressive mood” when 2 to 4 questions were answered with “more than half the time in the past two weeks”, also including one of the first two questions of the PHQ-9 questionnaire [24]. PHQ-9 was used as reference standard in this study because it has been shown to have a sensitivity of 88% and a specificity of 88% for major depression compared with diagnostic SCID interviews [24] as well as concurrent validity, high internal consistency, and test-retest reliability [25].

The single item screener “Have you been depressed or sad most of the past year?” uses a yes/no response format [11]. Based on a frequently used question for medical history taking, this single item question has been developed in the context of a randomised controlled trial of case finding for depression. The sample was predominantly female and Hispanic and was recruited at family and internal medicine clinics in the United States. Consecutive patients were randomly assigned to be asked the single item screener, to fill out the 20-item (CES-D), or to usual care. Corson et al. reported a LR + of the single item screener of 6.77 and an area under the ROC of .83 (95% confidence interval (CI) .79, .87) [20]. The single item screener was administered directly in advance to the PHQ-9.

### Statistical analyses

Firstly, the distribution of socio-demographic and clinical characteristics across the study sample was calculated for description of the study population. Secondly, we calculated several measures of test performance of the single item screener in comparison to the reference standard PHQ-9. This was done for the PHQ-9 “depressive mood” definition as well as for the “major depressive disorder” definition based on a 2×2 table (see Table 1). Specifically, we calculated the prevalence of persons with “depressive mood” and of “major depressive disorder”. Sensitivity (the proportion of persons having depression according to the PHQ-9 who test positive in the single item screener), specificity (the proportion of persons without the disease according to the PHQ-9 who test negative in the single item screener), receiver operating characteristic (ROC) area, the positive likelihood ratio (LR+, the probability of a person who has the disease according to the PHQ-9 and tests positive in the single item screener divided by the probability of a person who does not have the disease and tests positive), the negative likelihood ratio (LR-, the probability of a person who has the disease and tests negative divided by the probability of a person who does not have the disease and tests negative), the positive predictive value (PPV, the proportion of persons testing positive in the single item screener who have the disease), and the negative predictive value (NPV, the proportion of persons testing negative in the single item screener who do not have the disease) of the single item screener in comparison with either PHQ-9 depressive disorder definition were calculated, including 95% confidence intervals. The ROC is a graphical plot of the fraction of true positives out of the total actual positives (sensitivity) vs. the fraction of false positives out of the total actual negatives (1-specificity), at various threshold settings. The area under the ROC is a measure for test accuracy with a value of 1 representing a perfect test and an area of 0.5 representing a worthless test. These analyses were repeated stratified for age group (34–44, 45–54, 55–64, 65–74, 75–85 years) and gender (female, male), two variables known to be linked with a different prevalence of depressive disorders in the general population [26]. Additionally, the proportion of false positive test results was calculated using the PHQ-9 “depressive mood” definition. All analyses were done using STATA version 11.0 (Stata Corporation, College Station, Texas, USA).

**Table 1:** 2 × 2 table of the single item screener using the “depressive mood” definition and the “major depressive disorder” definition of the 9-item Patient Health Questionnaire (PHQ-9) as reference standard

	**Single item screener**		**Total**
**PHQ-9 “depressive mood”**	Positive	Negative	
Abnormal	406	475	881
Normal	130	2139	2269
**PHQ-9 “major depressive disorder”**			
Abnormal	169	35	204
Normal	367	2579	2946
Total	536	2614	3150

PHQ-9: 9-item depression module of the Patient Health Questionnaire.

## Results

Table 2 depicts the socio-demographic and clinical characteristics of the study sample. The proportion of male and female participants was almost equal with all age groups included being adequately represented. 21.63% of male participants and 33.93% of female participants were categorised to have “depressive mood” according to the established PHQ-9 definition. “Major depressive disorder” was prevalent in 4.46% of men and in 8.37% of women. The prevalence of depressive disorders of either definition increased with advancing age.

**Table 2:** Socio-demographic and clinical characteristics of the study sample

**Characteristic**	**Male**	**Female**	**Total**
	**N (%)**	**N (%)**	**N (%)**
All age groups	1,545 (48.52%)	1,639 (51.48%)	3,184 (100%)
34-44 years	320 (20.71%)	323 (19.71%)	643 (20.19%)
45-54 years	330 (21.36%)	404 (24.65%)	734 (23.05%)
55-64 years	372 (24.08%)	394 (24.04%)	766 (24.06%
65-74 years	323 (20.91%)	347 (21.17%)	670 (21.04%)
75-85 years	200 (12.94%)	171 (10.43%)	371 (11.65%)
Basic education	912 (59.22%)	968 (59.31%)	1880 (59.04%)
Diabetes mellitus	141 (9.16%)	117 (7.15%)	258 (8.84%)
Angina pectoris	112 (7.29%)	134 (8.20%)	246 (8.41%)
Previous hospitalisation due to myocardial infarction	58 (3.77%)	30 (1.83%)	88 (2.85%)
Intake of antidepressants	40 (2.60%)	101 (6.16%)	141 (4.64%)
PHQ-9 “depressive mood”, all age groups	330 (21.63%)	551 (33.93%)	881 (27.97%)
34-44 years	49 (15.41%)	79 (24.61%)	128 (20.03%)
45-54 years	57 (17.33%)	123 (30.60%)	180 (24.62%)
55-64 years	94 (25.41%)	146 (37.15%)	240 (31.45%)
65-74 years	78 (24.53%)	133 (38.66%)	211 (31.87%)
75-85 years	52 (27.23%)	70 (42.68%)	122 (34.37%)
PHQ-9 “major depressive disorder”, all age groups	68 (4.46%)	136 (8.37%)	204 (6.48%)
34-44 years	10 (3.14%)	14 (4.36%)	24 (3.76%)
45-54 years	14 (4.26%)	31 (7.71%)	45 (6.16%)
55-64 years	14 (3.78%)	32 (8.14%)	46 (6.03%)
65-74 years	13 (4.09%)	39 (11.34%)	52 (7.85%)
75-85 years	17 (8.90%)	20 (12.20%)	37 (10.42%)

PHQ-9: 9-item depression module of the Patient Health Questionnaire.

The prevalence of “depressive mood” increased from 20% (95% CI 17–23.3) in persons aged 34-to 44 years to 34% (29–39.4) in persons older than 75 years. Sensitivity of the single item screener was low across all age groups and genders, though it increased from 37.5% (29.1-46.5) to 52.5% (43.2-61.6) with advancing age. Specificity was >90% in all subgroups investigated, with very high values of >95% in persons younger than 55 years and in males. An area under the curve (AUC) of.702 (.685-.719) in the ROC analysis of the total sample was moderately good (Table 3).

**Table 3:** Prevalence and test performance of the single item screener using the “depressive mood” definition of the 9-item Patient Health Questionnaire (PHQ-9) as reference standard (95% confidence interval)

**Age group**	**Prevalence**	**Sensitivity**	**Specifity**	**ROC area**	**LR+**	**LR-**	**PPV**	**NPV**
34-44	20 (17–23.3)	37.5 (29.1-46.5)	96.5 (94.5-97.9)	.67 (.627-.713)	10.6 (6.42-17.7)	.648 (.556-.742)	72.7 (60.4-83)	86 (82.9-88.8)
45-54	25 (22–27.9)	46.1 (38.7-53.7)	95.5 (93.4-97)	.708 (.67-.745)	10.2 (6.72-15.4)	.565 (.493-.647)	76.9 (67.8-84.4)	84.4 (81.3-87.2)
55-64	31 (28–34.9)	45.8 (39.4-52.4)	93.7 (91.3-95.6)	.698 (.664-.731)	7.26 (5.08-10.4)	.578 (.514-.651)	76.9 (69.1-83.6)	79 (75.6-82.2)
65-74	32 (28–35.6)	47.9 (41–54.8)	92.7 (89.9-94.9)	.703 .667-.739)	6.54 (4.58-9.35)	.562 (.493-.642)	75.4 (67.2-82.4)	79.2 (75.4-82.6)
75-85	34 (29–39.4)	52.5 (43.2-61.6)	91 (86.6-94.3)	.717 (.669-.765)	5.82 3.74-9.05)	.523 (.432-.632)	75.3 (64.7-84)	78.5 (73.1-83.3)
Female	34 (32–36.3)	48.8 (44.6-53.1)	92.3 (90.5-93.8)	.705 (.683-.728)	6.31 (5.05-7.89)	.555 (.51-.603)	76.4 (71.6-80.8)	77.8 (75.4-80.1)
Male	22 (20–23.8)	41.5 (36.1-47)	96.1 (94.8-97.1)	.688 (.661-.715	10.6 (7.76-14.4)	.809 (.555-.667)	74.5 (67.5-80.6)	85.6 (83.6-87.5)
Total	28 (26–29.6)	46.1 (42.8-49.4)	94.3 (93.2-95.2)	.702 (.685-.719)	8.04 (6.71-9.64)	.572 (.538-.608)	75.7 (71.9-79.3)	81.8 (80.3-83.3)

ROC: receiver operating characteristic; LR+: positive likelihood ratio; LR-: negative likelihood ratio; PPV: positive predictive value; NPV: negative predictive value.

An LR + of >10 indicates that the post-test probability of having “depressive mood” is considerably increased. LR+ > 10 have been detected in our analysis in the younger age groups and in the male study population, but not in the higher age groups or in females, resulting in a LR + of 8.04 (6.71-9.64) for the total sample. LR- indicate the ability of the single item screener to decrease the post-test probability of having “depressive mood”, the conventional cut-point being LR- < .1. LR- in our analysis ranged from 0.523 (.432-.632) to 0.809 (.555-.667) indicating no reasonable decrease in post-test probability. PPVs correspond to a probability of having “depressive mood” in the presence of a positive single item screener of >70% in all subgroups investigated. NPVs ranging from 77.8% (75.4-80.1) to 86% (82.9-88.8) relate to fairly high probability to be healthy when the single item response is negative (Table 3). The proportion of false-positive test results (single item screener positive, but no diagnosis of “depressive mood” in PHQ-9) was 130/2269, i.e. 5.7%, ranging from 3.5% in the 34–44 age group up to 8.9% in the 75–85 age group.

When using the more conservative classification of PHQ-9, 6.5% (5.6-7.4) in the total sample were identified as having a “major depressive disorder” (3.8% (2.4-5.5) in the 34–44 age group, N = 24; 10% (7.4-14.1) in the >75 age group, N = 37). In comparison to this PHQ-9 definition, the single item screener demonstrated fairly good sensitivity with 75% (53.3-90.2) in the low-prevalence age group of 34–44 up to 86.5% (71.2-95.5) in those >65 years of age. Specificity of 87.5% (86.3-88.7) in the total sample was also fairly good with comparably low specificity in those subgroups with comparably high sensitivity and vice versa (e.g. specificity of 92.2% (89.8-94.2) in the 34–44 age group and 83.3% (78.8-87.3) in the >75 age group). As compared to the “depressive mood” definition, using the “major depressive disorder” definition resulted in a significantly higher ROC area of .852 (.825-.879) (Table 4).

**Table 4:** Prevalence and test performance of the single item screener using the “major depressive disorder” definition of the 9-item Patient Health Questionnaire (PHQ-9) as reference standard (95% confidence interval)

**Age group**	**Prevalence**	**Sensitivity**	**Specifity**	**ROC-area**	**LR+**	**LR-**	**PPV**	**NPV**
34-44	3.8 (2.4-5.5)	75 (53.3-90.2)	92.2 (89.8-94.2)	.836 (.747-.925)	9.61 (6.73-13.7)	.271 (.136-.542)	27.3 (17–39.6)	99 (97.7-99.6)
45-54	6.2 (4.5-8.2)	86.7 (73.2-94.9)	89.9 (87.2-92.1)	.883 (.832-.935)	8.62 (6.7-11.1)	.148 (.070-.312)	36.1 (27.1-45.9)	99 (97.9-99.6)
55-64	6 (4.4-8.0)	76.1 (61.2-87.4)	84.9 (82.1-87.5)	.805 (.741-.869)	5.05 (3.98-6.41)	.282 (.168-.472)	24.5 (17.7-32.4)	98.2 (96.8-99.1)
65-74	7.9 (5.9-10.2)	86.5 (74.2-94.4)	85.4 (82.4-88.1)	.86 (.811-.909)	5.93 (4.76-7.39)	.158 (.0791-.314)	33.6 (25.7-42.2)	98.7 (97.3-99.5)
75-85	10 (7.4-14.1)	86.5 (71.2-95.5)	83.3 (78.8-87.3)	.849 (.79-.909)	5.19 (3.93-6.84)	.162 (.0717-.367)	37.6 (27.4-48.8)	98.1 (95.7-99.4)
Female	8.4 (7.1-9.8)	81.6 (74.1-87.7)	83.8 81.8-85.6)	.827 (.973-.861)	5.04 (4.38-5.8)	.219 (.154-.313)	31.5 (26.7-36.7)	98 (97.1-98.7)
Male	4.5 (3.5 -5.6)	85.3 (74.6-92.7)	91.4 (89.8-92.8)	.883 (.84-.926)	9.87 (8.13-12)	.161 (.0908-.285)	31.5 (24.9-38.8)	99.3 (98.6-99.6)
Total	6.5 (5.6-7.4)	82.8 (77–87.7)	87.5 (86.3-88.7)	.852 (.825-.879)	6.65 (5.93-7.46)	.196 (.145-.265)	31.5 (27.6-35.7)	98.7 (98.1-99.1)

ROC: receiver operating characteristic; LR+: positive likelihood ratio; LR-: negative likelihood ratio; PPV: positive predictive value; NPV: negative predictive value.

The single item screener is not useful for ruling in major depressive disorder, as the LR + in the total sample is 6.65 (5.93-7.46) and for most subgroups far away from >10. The ability of ruling out major depressive disorder is much better with a LR- of .196 (.145-.265) in the total sample. However, in none of the subgroups investigated, the LR- was < .1. Given the low prevalence of major depressive disorder (according to PHQ-9), the PPVs and NPVs as shown in Table 4 must be interpreted with care, as a prevalence of >15% is considered to be adequate for this type of analysis. Albeit, PPVs of about 30% indicate a quite low probability of having major depressive disorder in the presence of a positive single item screener (resulting a high number of false positives), whereas it is almost sure that a person does not have a major depressive disorder in the presence of a negative test result (NPV in the total sample 98.7% (98.1-99.1)).

## Discussion

Interpreting the clinical meaning of the test result of a simple yes/no single item question (“Have you been depressed or sad most of the past year?”) in comparison to the 9-item PHQ instrument is complex: In the presence of a positive test result, the likelihood of the person having a clinically relevant depressive disorder is considerably increased (LR + 8.04 in comparison to PHQ-9 “depressive mood”, LR + 6.65 in comparison to PHQ-9 “major depressive disorder”). A person presenting with a positive single item screener would therefore be in need for a more detailed evaluation of depressive symptoms. In the presence of a negative test result, a major depressive disorder is relatively unlikely (LR- 0.196 in comparison to PHQ-9 “major depressive disorder”), though the presence of a major depressive disorder cannot completely excluded. However, a negative test result does only minimally decrease the likelihood of a person having depressive mood (LR- 0.572 in comparison to PHQ-9 “depressive mood”). As a result of the varying prevalence of depressive disorders across age groups and between females and males, we detected differences in test performance measures across these strata. However, the differences were not clear enough to recommend the single item for specific use in certain groups of patients.

When associating this study with previous research, our results for sensitivity (83% in comparison with PHQ-9 “major depressive disorder”) are comparable with Williams et al. [11] (85%) and slightly higher than those of Corson et al. (78%) [20]. With respect to specificity, the present study (88%) and the results of Corson et al. (88%) are concordant, both done in comparison with PHQ-9 “major depressive disorder”. However, when Williams et al. investigated the single item screener in comparison to SCID interviews specificity was considerably lower (66%). Given the fact that the PHQ-9 has been shown to have a specificity of 80-90% in comparison to SCID interviews, [24] the previous findings seem plausible.

However, poor specificity as compared to the gold standard translates into high rates of false-positive test results. There is a vivid discussion on whether current criteria for clinical diagnosis of depression are medicalising sadness [27] or whether - in contrary - there are still many people missing on life saving treatment [28]. The debate also includes whether screening for depression increases over diagnosis or whether it is an effective public health measure [14,29]. We did not detect substantial differences in the rates of false-positives between the single item screener and PHQ-9 (5.7% of single item test results in comparison to PHQ-9 “depressive mood”). However, as stated above, we did not compare against the gold standard, and there is a considerable amount of false-positives when applying the PHQ-9 which we were not able to detect in the present study [22,24,25].

In comparison to PHQ-9, the main limitation of the single item screener is the relatively low ability to detect less-than-severe depressive disorders. Therefore, the utility of the single item in clinical context is very limited. It might be used as a first step of a screening procedure in combination with other, more detailed assessment instruments. For example, such a two-step screening procedures has been recommended by the American Heart Association for patients with coronary heart disease [30]. Elderon et al. evaluated this recommendation using the PHQ-2 and the PHQ-9 sequentially and found this procedure to be highly specific, poorly sensitive, but predictive of poor coronary outcomes [31]. Similar two-step screening procedures may also be applied in other settings or other patient populations.

In contrast to clinical settings, the single item screener may be helpful for selection of specific patient populations if the absence of a depressive disorder (negative test result) or the presence of a major depressive disorder (positive test result) is selection criterion and if space, time or resources for more comprehensive questionnaires are limited.

When interpreting this study, several limitations need to be considered. This is a secondary analysis of data of the large, population-based KORA cohort study which has not specifically been designed for the research question addressed in the present manuscript. SCID interviews which were not available in this project are considered to be the gold standard for diagnosing depressive disorders in research contexts. However, we used PHQ-9 as the reference standard which has been shown to have good concordance with clinical diagnosis of depression [32]. Additionally, all participants lived in Bavaria so that there may be cultural differences in the prevalence and diagnostic identification of depressive disorders as compared to other countries. Moreover, some of the persons who were eligible for the study were not willing to participate (S3 baseline survey response rate = 74.9%), and some of those who participated at baseline, had dropped out for the F3 follow-up (F3 follow-up survey response rate: 76% of S3 participants) so that selection bias cannot be excluded. However, the demographic and socioeconomic characteristics of the underlying population roughly reflect those of the average middle European population in general [23]. In addition, the reader should keep in mind that the PHQ-9 assesses depressive symptoms within the last 2 weeks, whereas the single item screener inquires about the past year. So, the PHQ-9 is in line with a diagnosis of depression according to the DSM-IV or DSM-V criteria, when the single item screener includes a global assessment of a much longer interval but does not inquire detailed aspects of depression. Another limitation is that reliability of the single item screener, e.g. test-retest performance has not been evaluated so far and should be included in future research.

## Conclusions

In comparison to PHQ-9, the single item screener proposed by Williams et al. is able to moderately decrease the likelihood of major depressive disorders and to identify populations that should undergo additional, more detailed depression screening measures. However, in comparison to PHQ-9 the single item screener has a low ability to detect less-than-severe depressive disorders and can therefore not be recommended for routine use as a screening tool in clinical practice.

## Competing interests

The authors declare that they have no competing interests.

## Authors’ contributions

EB and MS designed the study and drafted the manuscript. EB performed the statistical analyses. MEL and KHL reviewed the study design and helped to interpret data and to draft the manuscript. All authors read and approved the final manuscript.

## Pre-publication history

The pre-publication history for this paper can be accessed here:

http://www.biomedcentral.com/1471-2296/14/198/prepub
